# Primary and Recurrent Intraosseous Adenoid Cystic Carcinoma—Analysis of Two Cases and Literature Review

**DOI:** 10.3390/medicina60010100

**Published:** 2024-01-05

**Authors:** Chenlu Xu, Wenyi Shen, Yangxi Cheng, Dan Yu, Huiyong Zhu

**Affiliations:** 1 Department of Oral and Maxillofacial Surgery, The First Affiliated Hospital, Zhejiang University School of Medicine, Hangzhou 310003, China; zdxcl@zju.edu.cn (C.X.); wyshen@zju.edu.cn (W.S.); ChengYangxi@zju.edu.cn (Y.C.); 2 Department of Stomatology, Zhejiang University School of Medicine, Hangzhou 310058, China

**Keywords:** intraosseous adenoid cystic carcinoma, clinical, radiologic, pathological, immunohistochemical, prognosis

## Abstract

Adenoid cystic carcinoma (ACC) is a rare malignant tumor that mostly occurs in minor glands, especially in the palate. Intraosseous adenoid cystic carcinoma (IACC) is rarer. There is no clear conclusion on the clinical, radiologic and pathological characteristics of IACC because of few reported IACC cases, leading to insufficient understanding of IACC. We reviewed 52 previous reports of primary IACC (PIACC) and analyzed the clinical features of those patients involved, attempting to provide a better understanding of PIACC. Moreover, we present a case of primary PIACC and a case of recurrent IACC (RIACC). The two patients showed similarities in clinical and pathological results, along with slight differences in radiological and immunohistochemical results. The patient of case 1 seemed to display a worse prognosis, which can only be proved after long term follow-up.

## 1. Introduction

Adenoid cystic carcinoma (ACC) is a rare malignant tumor that originates from secretory glands such as the three major salivary glands, oral minor salivary glands, lacrimal glands, the breast and prostate and secretory tissue in paranasal sinuses, the larynx and the trachea [1]. ACC accounts for only 1% of all malignant neoplasms of the head and neck, but it can account for up to 30% of malignant salivary gland tumors [2]. ACC is an indolent tumor, showing a slow growth pattern, with a 5-year survival rate of 80–85% [3]. Lymph node metastasis rarely occurs, with an approximate rate of 17–37% [4]. However, the characteristics of perineural invasion (PNI) and hematogenous metastasis of ACC determine the high occurrence rate of distant metastasis (DM), 16.1–72.7% approximately [5]. The most common organ of DM is the lung, accounting for 74.5–94.4%, followed by the liver, bone and others [6]. Young, A. et al. [7] found that despite active treatment, ACC patients still exhibit a local recurrence (LR) rate of 15–85% and a DM rate of 25–55% after 5 years. Due to the LR and DM of ACC, the 10-year survival rate is only 50–60%, and the 15-year survival rate is as low as 30–35% [3,5].

Pathological results of ACC are highly correlated with prognosis. ACC of a solid predominant pattern is related to poor prognosis. To further evaluate the pathological results and prognosis of ACC patients, Perzin and Szanto divided ACC into three grades: tubular dominated with no solid pattern (grade I), cribriform predominated with solid component proportion < 30% (grade II) and solid component proportion > 30% (grade III) [8,9]. Spiro also divided ACC into three grades: mostly tubular or cribriform (grade I), solid component proportion > 50% (grade II) and only solid (grade III) [10]. Considering the significant researcher bias in the statistical analysis of the percentage of the solid tumor component, van Weert simplified the pathological grading of ACC, dividing ACC into grade I (without solid component) and grade II (with solid component) [11]. But van Werert did not provide a clear definition of “solid”. Naruhiko Morita et al. [12] defined a solid tumor nest as tissue composed of tumor cells with no recognizable duct lumen or cystic space under low-power magnification, tried to estimate the maximum oval fitting the largest solid tumor nest and divided ACC by the length of the minor axis of the oval. The MinAmax (minor axis maximum) grading system provided by Naruhiko divides ACC into two grades: MinAmax ≤ 0.2 mm (grade I) and MinAmax > 0.2 mm (grade II). According to Naruhiko Morita, the MinAmax system has the minimum interobserver variability, which is more convincing in prognostic assessment.

Intraosseous adenoid cystic carcinoma (IACC) is rarer, with a total number of reported cases to date less than 0.4% of all ACC cases [13]. Since 1955, only 52 cases of primary IACC (PIACC) have been reported with relatively complete data, but patients of recurrent IACC (RIACC) have seldom been reported, resulting in incomplete understanding of IACC. Moreover, due to the lack of specificity of radiologic and clinical manifestations, IACC is often misdiagnosed. Here, we report a case of PIACC and a case of RIACC. Two patients shared similarities in clinical and radiological characteristics. We reviewed all publicly reported PIACC and summarized clinical, radiological and pathological manifestations of PIACC. In addition, we compared the medical history, radiological results, pathological results and immunohistochemical results of the two patients in our report, analyzed the prognostic factors and attempted to provide perspectives on distinguishing between PIACC and RIACC.

## 2. Case Report

### 2.1. Case 1

**Figure 1:**
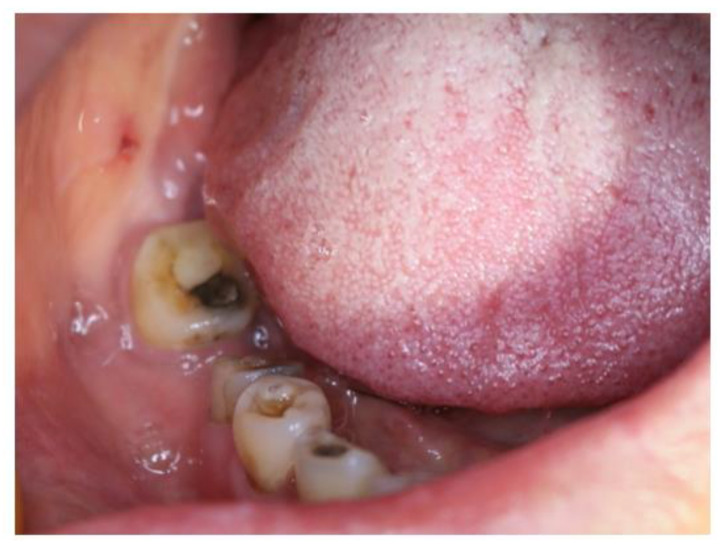
Extraoral examination showed swelling on the buccal side of the right mandibular second molar while the overlying mucous was normal.

A 65-year-old woman was reported to the First Affiliated Hospital, College of Medicine, Zhejiang University, for further examination in July, 2022, with the complaint of pain and swelling in the lower right back teeth region. After extraction of the right mandibular first molar 6 months ago in a local hospital, swelling on the lower right back teeth region gradually expanded with persistent pain. A biopsy in the local hospital indicated ACC. The patient exhibited paresthesia of the right lower lip on clinical examination, with no symptoms of trismus. Extraoral examination showed a nontender and bony hard swelling on her buccal side of the right mandibular second molar (Figure 1). Tooth #46 was missing and the remaining teeth showed no mobility. The overlying mucus was normal with no red or ulcer. The patient was in good health before.

**Figure 2:**
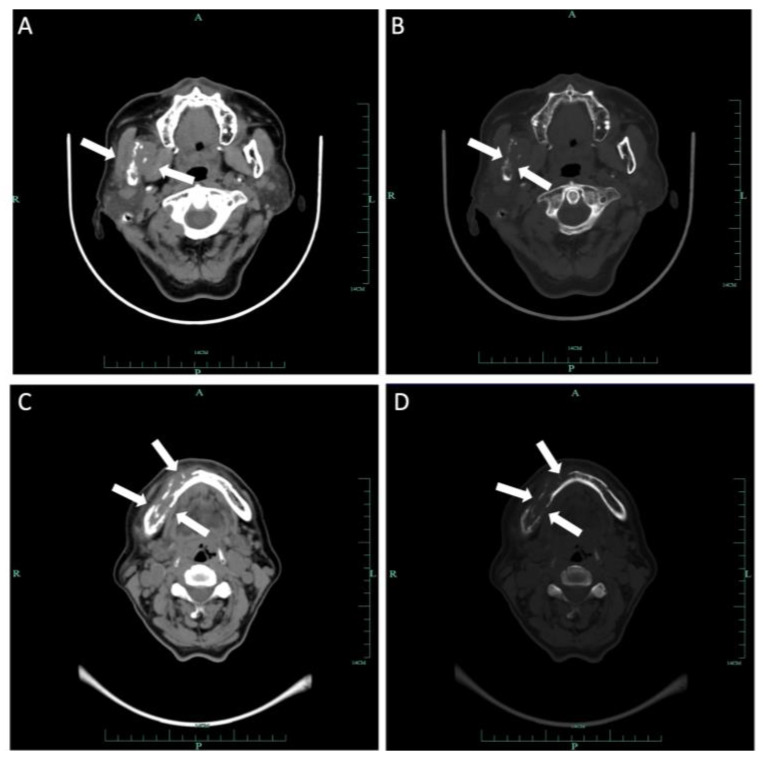
Axial CT of soft tissue (**A**) and bone windows (**B**) demonstrated a soft tissue mass on the body, angle and ramus of right mandibular involving the mandibular canal with extension to adjacent muscles. Additional axial soft tissue (**C**) and axial bone window (**D**) images demonstrated expansion of the mass to the body of the left mandibular. Wormlike bone resorption of buccal and lingual bone cortex of the right mandible can be seen.

**Figure 3:**
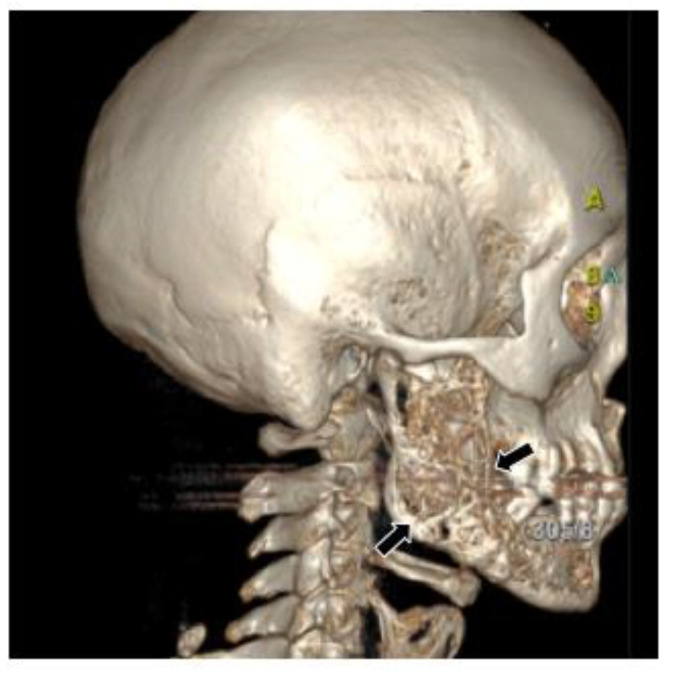
Three-dimensional reconstruction image showed honeycomb-like bone destruction of the buccal bone cortex.

**Figure 4:**
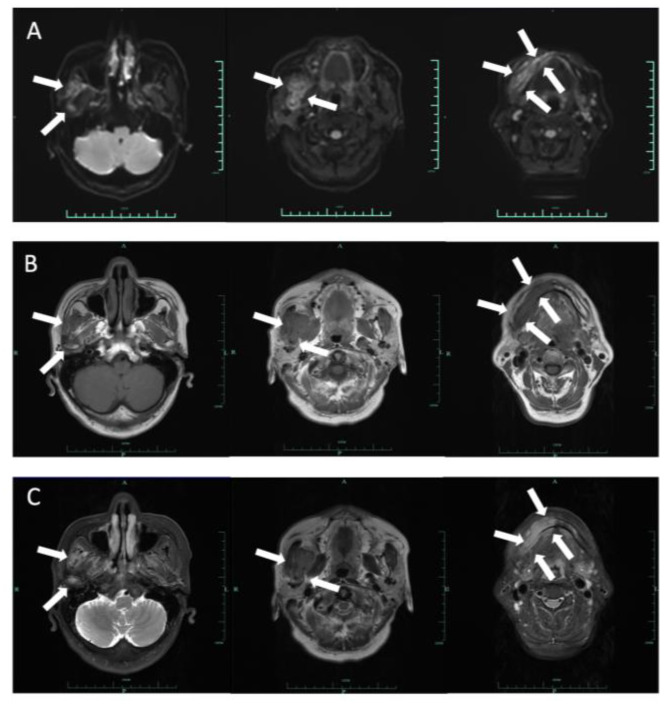
Axial DWI (**A**), T1-weighted (**B**) and T2-weighted (**C**) images demonstrated a 6.8 × 2.6 cm lesion on the right side from the body of mandibular to the condylar neck involving the mandibular canal, the right internal pterygoid and masseter muscles. The mass extended slightly to the left mandibular anterior tooth area, with an ill-defined margin. This lesion is hypointense on T1 and hyperintense on T2 and DWI.

**Figure 5:**
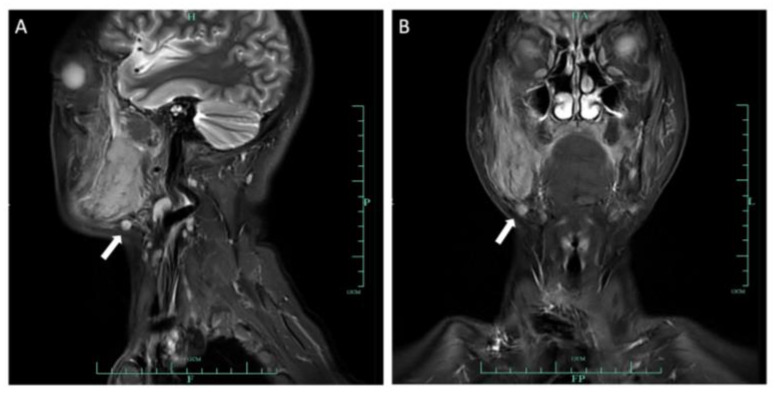
Sagittal (**A**) and coronal (**B**) MRI images demonstrated an enlarged lymph node in the right submandibular area, with a diameter of 0.8 cm.

Computed tomography (CT) and three-dimension reconstruction images showed an ill-defined radiolucent lesion involving the right side of the body, angle and ramus of the mandible, with a honeycomb-like bone destruction of the bone cortex (Figure 2 and Figure 3). On magnetic resonance imaging (MRI) examination, a proton density image and T2-weighted image (T2WI) showed a hyperintense region on the right side from the mandibular body to the condylar neck involving the mandibular canal, the right internal pterygoid and masseter muscles, approximately 6.8 × 2.6 cm in size, compared with the left side (Figure 4). An enlarged lymph node can be seen in the right submandibular area, with a diameter of 0.8 cm (Figure 5). No obvious abnormal findings were found in the surrounding salivary glands, including the three major glands and other minor glands. The nasal and paranasal sinuses, nasopharynx and larynx were normal as well. In addition, chest CT did not point out pulmonary metastasis.

**Figure 6:**
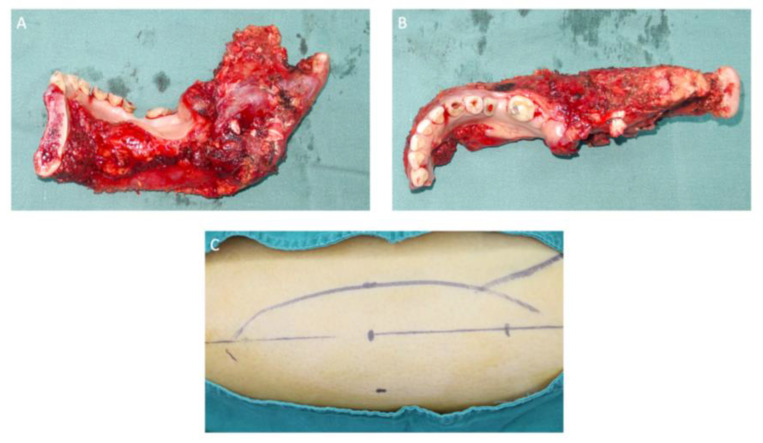
Sagittal (**A**) and axial (**B**) views of the mandibular section specimen. The mandibular section specimen included the entire body, the angle, the condyle and the coronoid process of the right mandible and partial body of the left mandible. (**C**) A design of the anterolateral thigh flap with a size of 13 × 6 cm.

Under general anesthesia, the mass ranging from the left mandibular canine to the right condyle was surgically removed (Figure 6). Right radical neck dissection with lymph nodes (Levels I–III) and anterolateral thigh flap reconstruction were performed at the same time.

**Figure 7:**
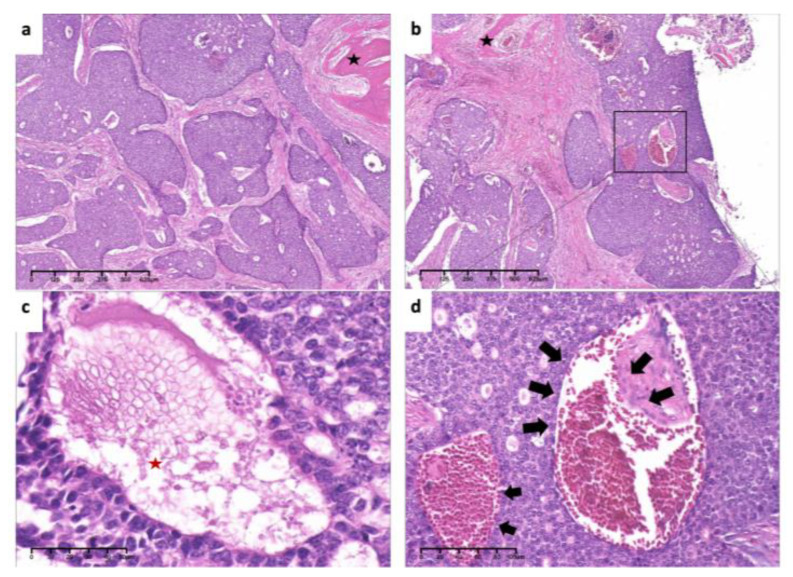
Pathological findings. (**a**). Tumor cells arranged mainly in solid nests, surrounded by the bone (black asterisk) (×4, H&E. Bar: 125 μm). (**b**). Low-power view of invasion and encirclement of vascular (×4, H&E. Bar: 125 μm). (**c**). High-power view of necrosis (red asterisk), hyperchromasia and cellular atypia (×40, H&E. Bar: 10 μm). (**d**). Invasion of tumor cells inside vascular (arrows) (×20, H&E. Bar: 20 μm). H&E: Hematoxylin and eosin.

**Figure 8:**
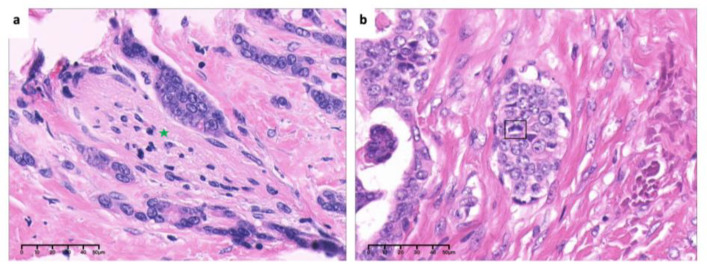
High-power view of (**a**). invasion and encirclement of nerve (green asterisk) and (**b**). mitosis of tumor cells (box) (×40, H&E. Bar: 10 μm). H&E: Hematoxylin and eosin.

**Figure 9:**
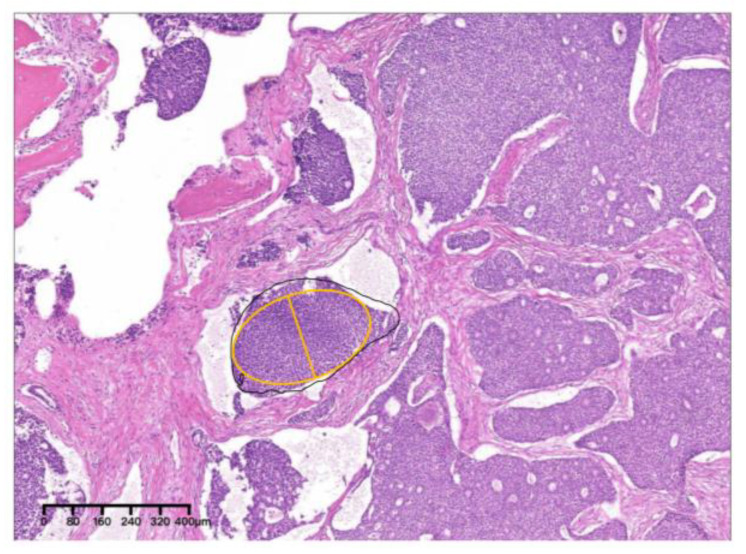
The solid nest indicated by the yellow oval was the largest, with a MinAmax of 0.225 mm (×4, H&E. Bar: 80 μm). H&E: Hematoxylin and eosin.

Postoperative histopathological examination showed that the tumor cells were arranged in a solid predominant pattern, with several nests showing a tubular and cribriform pattern (Figure 7). Pathological examination revealed one metastatic lymph node. PNI was observed (Figure 8). Cellular atypia, mitotic figures and necrosis can be noticed. Pathological grade was determined as grade II according to the MinAmax grading system (MinAmax = 0.225 mm) (Figure 9). All surgical margins were pathologically negative. Immunohistochemistry results were as follows: SMA (weak +), CK (pan) (+), CD117 (+), CD43 (−), s-100 (−), calponin (+) and p63 (+). Based on these findings, the patient was clinically diagnosed with PIACC (T4aN1M0).

The patient received postoperative radiotherapy (PORT) and a 15-month follow-up showed no evidence of recurrence or metastasis.

### 2.2. Case 2

**Figure 10:**
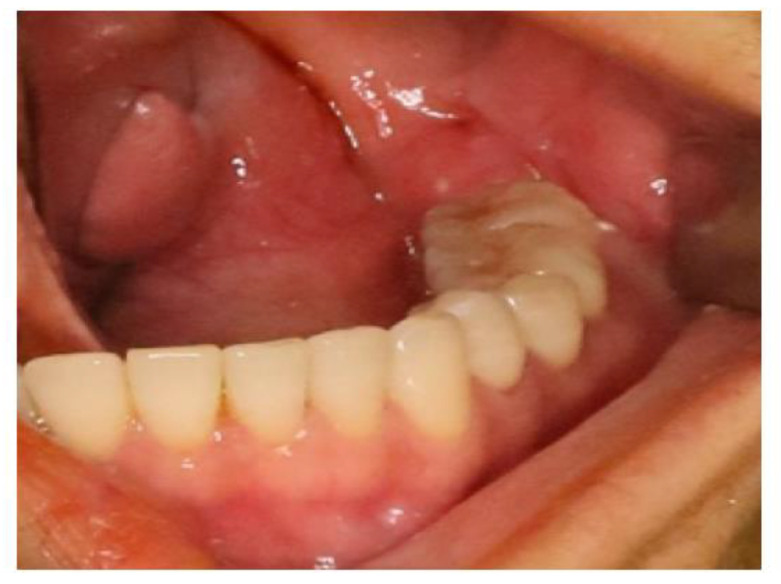
Extraoral examination showed no abnormal signs. The gingival mucosa was clear and normal with no red or swelling.

**Figure 11:**
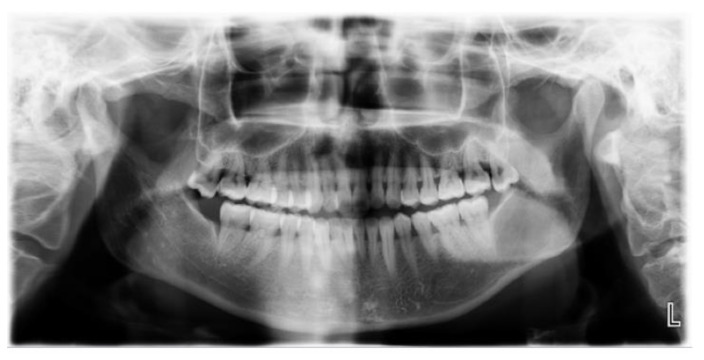
Panoramic radiograph imaging revealed a cystic lesion on the left mandible ramus with ill-defined margin infiltrating to the mandibular canal.

**Figure 12:**
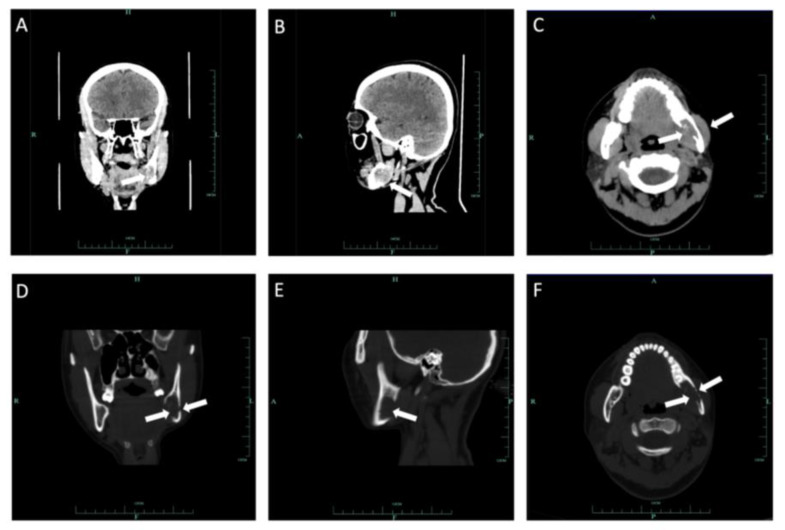
Coronal CT soft tissue (**A**), sagittal soft windows (**B**) and axial soft windows (**C**) demonstrated a soft tissue mass on the ramus of the left mandible involving the left mandibular canal. Coronal CT bone tissue (**D**), sagittal bone windows (**E**) and axial bone windows (**F**) demonstrated defection of lingual bone cortex and perforation of buccal bone cortex.

**Figure 13:**
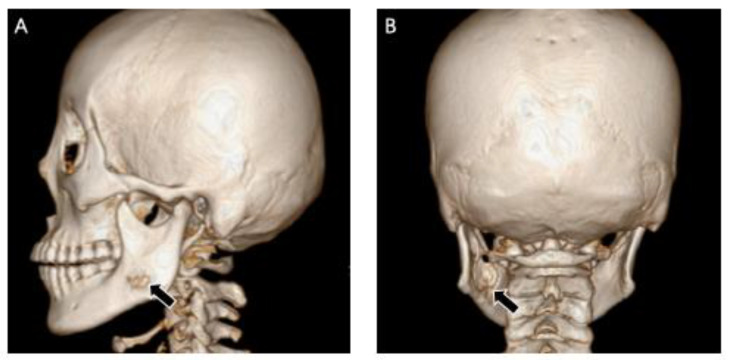
Sagittal image (**A**) and coronal image (**B**) of three-dimension reconstruction showed bone destruction of the lingual and buccal bone cortex on the left ramus of the left mandible.

In September 2022, a 35-year-old woman was referred to the First Affiliated Hospital, College of Medicine, Zhejiang University, with the main complaint of paresthesia of the left lower lip. The patient reported left submandibular gland resection 14 years ago with a pathology result showing ACC at that time. Radiotherapy with a total dose of 70 Gy was received by the patient after the initial surgery. Extraoral examination showed no abnormal signs (Figure 10). Panoramic radiograph imaging showed a cystic lesion on the ramus of the left mandible (Figure 11). CT and three-dimensional reconstruction images revealed a thin and perforated buccal cortical bone and a destroyed lingual cortical bone on the left side of mandibular ramus involving the left mandibular canal (Figure 12 and Figure 13). An initial diagnosis of primary intraosseous malignant tumor or recurrent intraosseous ACC was considered.

**Figure 14:**
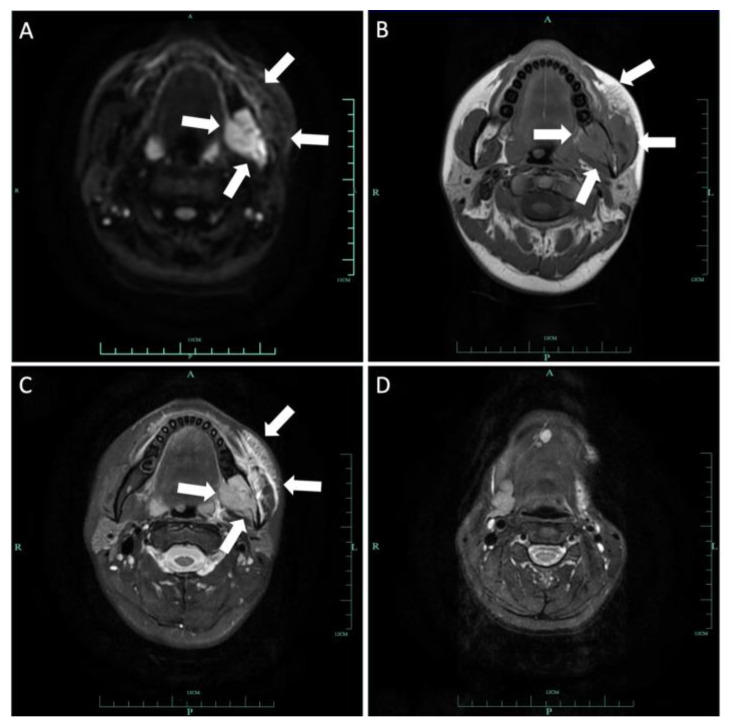
Axial DWI (**A**), T1-weighted (**B**) and T2-weighted (**C**) images demonstrated a 2.4 × 2.9 cm lesion on the left mandibular ramus involving the mandibular canal. The mass invaded the left pterygopalatine fossa and parapharyngeal space inward and extended outward to the left masseter muscle space. Patchy signal in the parapharyngeal space and the masticatory muscle space can be seen. This lesion is hypointense on T1 and hyperintense on T2 and DWI. Axial T2-weighted (**D**) image demonstrated loss of left submandibular gland.

**Figure 15:**
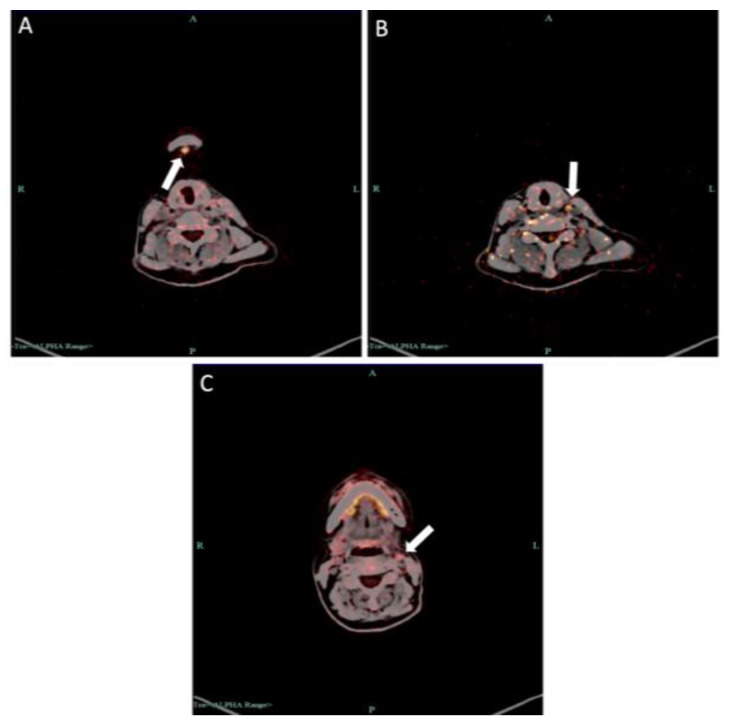
PET–CT examination showed increased SUV in (**A**) submental lymph node, (**B**) lymph nodes in deep surface of left sternocleidomastoid muscle and (**C**) left cervical parasheath lymph nodes.

For further evaluation of the left mandibular ramus, MRI was performed. A hyperintense region on the left mandibular ramus was observed on a proton density image and a T2-weighted image (T2WI) with an ill-defined boundary, approximately 2.4 × 2.9 cm in size (Figure 14). Lymph node enlargement was detected near the left cervical sheath, in the deep surface of the left sternocleidomastoid muscle and in the left submental region, with a largest diameter of 0.7 cm (Figure 15). There were no abnormal signs in bilateral parotid glands, bilateral sublingual glands or the right submandibular gland. Positron emission tomography (PET)–CT showed an increase in 18F-fluorodeoxyglucose (FDG) accumulation in the left mandible with a maximum standardized uptake value (SUV) of 4.2. Adjacent soft tissue had a maximum SUV of approximately 8.9. Lymph nodes in the left cervical sheath, deep surface of the left sternocleidomastoid muscle and submental region had a maximum SUV of approximately 3.9. PET–CT and chest CT showed no metastatic signs in the lungs.

**Figure 16:**
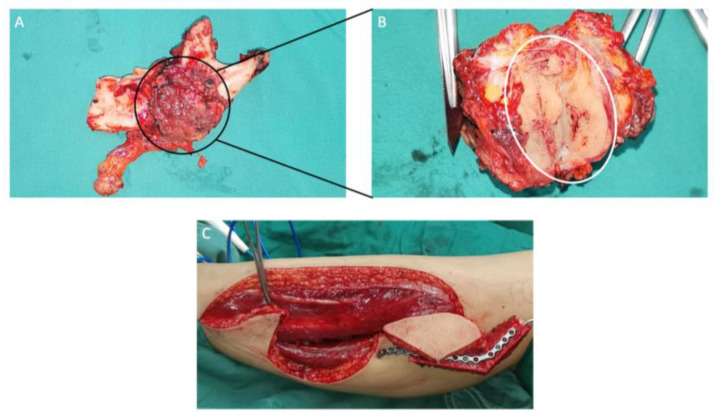
(**A**) Mandibular resection specimen included the partial body, the angle, the condyle and the coronoid process of the left mandible. (**B**) Muscle groups and the tumor tissue (white circle) attached to the inner surface of the left mandible. (**C**) A design of the free fibula flap.

Biopsy was performed under local anesthesia. Part of the left mandible was incised and ACC was suggested by pathological examination. The mass in the left mandibular ramus was then surgically removed under general anesthesia. Right partial mandibulectomy (Figure 16), radical neck dissection (levels I–III), plate reconstruction and free fibula osteomyocutaneous flap reconstruction were simultaneously performed.

**Figure 17:**
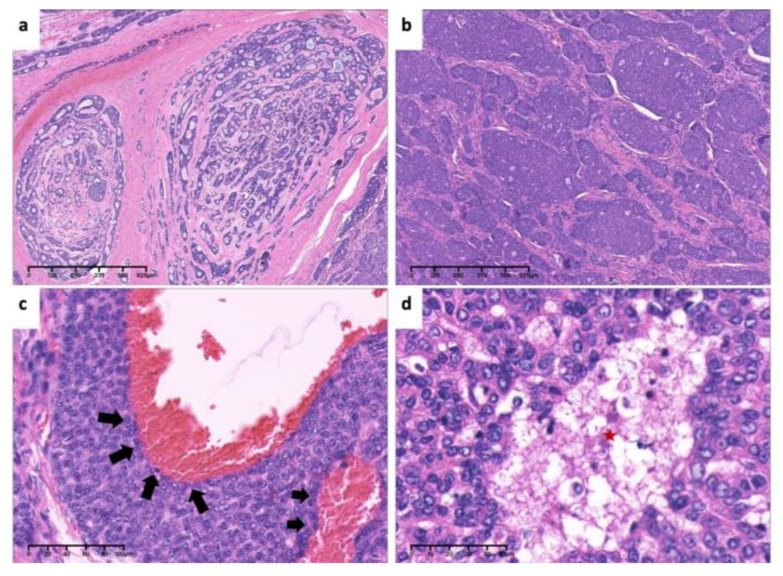
Pathological findings. (**a**). Tumor cells arranged in tubular and cribriform pattern (×4, H&E. Bar: 125 μm). (**b**). Tumor cells arranged in solid nests (×4, H&E. Bar: 125 μm). (**c**). Invasion of tumor cells in vascular wall(arrows) (×20, H&E. Bar: 20 μm). (**d**). High-power view of necrosis (red asterisk) and nuclear atypia (×40, H&E. Bar: 10 μm). H&E: Hematoxylin and eosin.

**Figure 18:**
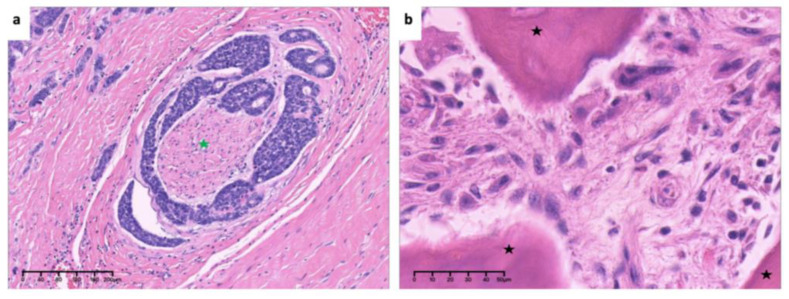
Low-power view of (**a**). invasion and encirclement of nerve (green asterisk) (×10, H&E. Bar: 40 μm) and high-power view of (**b**). hyperchromasia and cellular atypia of tumor cells around bone (black asterisk) (×40, H&E. Bar: 10 μm). H&E: Hematoxylin and eosin.

**Figure 19:**
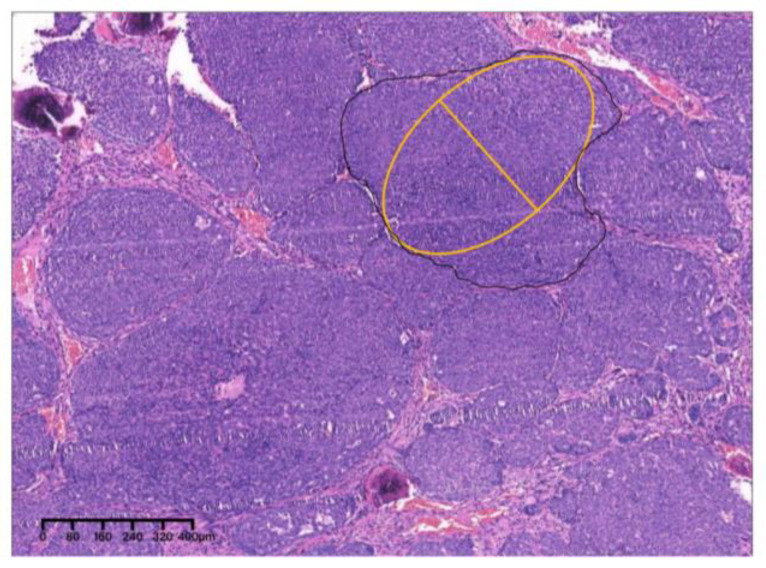
The solid nest indicated by the yellow oval was the largest, with a MinAmax of 0.404 mm (×4, H&E. Bar: 80 μm). H&E: Hematoxylin and eosin.

Pathologically, the tumor cells were arranged in a solid and cribriform pattern. Nucleoli were clear with visible mitotic figures (Figure 17). PNI can be seen (Figure 18). Pathological grade was determined as grade II according to the MinAmax grading system (MinAmax = 0.404 mm) (Figure 19). All surgical margins were negative. Immunohistochemistry results were as follows: CK (pan) (+), CD117 (+), CK7 (+), Ki-67 (+, 30%), s-100 (+), calponin (−) and p63 (−). RIACC (T4aN2aM0) was diagnosed.

The patient did not receive any adjuvant therapy after surgery and a 14-month follow-up showed no evidence of recurrence or metastasis.

## 3. Literature Review of PIACC

### 3.1. Materials and Methods

A literature search was performed on PubMed, Embase Ovid, the Cochrane Library and Science Direct. The medical subject headings were carcinoma, adenoid cystic or cylindroma combined with mandible. Only articles published in English were included. Articles related to PIACC were selected. Since 1955, there have been only 52 publicly reported cases of PIACC, with case 1 being the 53rd case (Table 1). Among all selected articles, Hu et al. [14] summarized a total of 47 PIACC cases before 2017, while Indu [15], Reddy [16], Savithri [17], Shome [18] and Sasaki [19] reported the 48th, 49th, 50th, 51st and 52nd case, respectively.

### 3.2. Results

Hu et al. [14] found a female-to-male ratio of 1.1:1 (24 women and 21 men), showing no significant differences between sexes in PIACC patients. By 2023, the female-to-male ratio of 53 PIACC patients had reached 1.2:1 (29 women and 24 men), showing a slight dominance of female patients. The age of patients ranged from 24 to 82 years old, with a peak prevalence in the fourth to sixth decades, in accordance with the report of Hu, with the mean age ranging from 52.8 year to 53.4 year. The majority of lesions occurred in the right mandible (19 cases on the right and 13 cases on the left). In addition, there was a clear predominance of PIACC localized in the body of the mandible (75.5%). It is worth noting that five cases involved the body, angle and ramus of the mandible, while one case only occurred in the right temporomandibular joint. Swelling, pain and paresthesia were the most common clinical manifestations in PIACC patients (56.6%, 52.8%, 26.4% respectively). In addition, seven (13.2%) patients showed tooth mobility, and four (7.5%) patients showed trismus. Case 1 was a 65-year-old female patient with a lesion involving the body to the ramus of the right mandible. The patient initially received tooth extraction treatment due to pain in the right back teeth region. But a proliferation of mass later developed in the extraction socket that quickly spread to the surrounding gums. The patient’s symptoms corresponded to previous reports.

Hu et al. [14] analyzed the radiologic features of 33 patients with complete imaging data before 2017 and found that 32 patients (97%) showed radiolucent destruction of bone, with 57.6% of patients having poorly defined boundaries. Only Favia et al. [20] reported a case of PIACC with mixed density in the apical region. Based on the study of Hu et al. and five newly published cases since 2017, we found that the imaging of PIACC often presents as a poorly defined osteolytic image centered on the mandible, with intact, thin or perforated bone cortex, consistent with radiological manifestations of case 1. It is often difficult to distinguish ACC from other malignant tumors in the jawbone based solely on imaging findings. In this situation, misdiagnosis often occurs. Among the 53 patients, definitive initial diagnosis was listed in 31 cases, with 7 cases misdiagnosed as ameloblastoma, 11 cases misdiagnosed as odontogenic infection or cyst, 1 case misdiagnosed as centricity carcinoma and 1 case misdiagnosed as periapical cervical dysplasia (Table 2). The imaging of case 1 showed a typical poorly defined osteolytic feature, with expansion of the mass and bone destruction in the buccal and lingual bone cortex. Comparing the bilateral mandibular ramus in case 1, a clear image of the mandibular nerve canal can be seen on the left side, while the mandibular nerve canal of the right mandible was completely occupied by the tumor. Additionally, due to the micro infiltration of ACC, the boundary of the lesion on the MRI imaging does not fully represent the actual boundary of the mass. The surgical margin of the tumor still needs to be determined based on the color and texture of the tissue.

Among the 53 patients, 10 cases had preoperative lymph node metastasis, 9 cases presented with DM at first visit and 3 cases showed both lymph node metastasis and DM. A total of 42 patients underwent surgical treatment, and 18 patients received simultaneous mandibular reconstruction. Among the 42 surgical patients, 28 patients continued to receive PORT, and 3 patients received both postoperative radiotherapy and chemotherapy. Due to imaging confirmation of lymph node metastasis, the patient of case 1 underwent bilateral partial mandible resection and right scapular hyoid neck lymph node dissection. Postoperative pathology also confirmed one metastatic lymph node. Because of a high possibility of LR, simultaneous mandibular reconstruction was not recommended. However, the patient had a high demand for appearance, so an anterior femoral skin flap and titanium plates were ultimately used to restore the appearance. Many studies have confirmed the positive role of PORT in local control [21,22,23]. So, PORT was recommended to reduce LR.

Among the 42 cases with specific pathological results, most PIACC patients exhibited a tubular and cribriform type (61.9%) while a portion of patients exhibited a solid type (38.1%). The pathological results of case 1 showed a solid predominant pattern, which was grade II in the van Weert system and grade II in the MinAmax system (0.225 mm). The local necrosis, increased mitotic rate, cellular atypia and more abundant cytoplasm of tumor cells prompted the high-grade transformation [24]. All these pathological characteristics of case 1 suggest a poor prognosis for the patient, which also reminds us of the necessity for close postoperative follow-up. Notably, Li et al. [25] found that ACC with poorly defined boundaries and bone destruction tends to exhibit pathological solid types, consistent with the radiological and pathological results of case 1.

The incidence of PNI in ACC is 48–82% [26] and predicts a poorer prognosis and lower survival rate. Research of Lukšić I et al. [27] showed that the disease-specific survival (DSS) rate could reach 90% for patients without PNI. However, for patients with PNI, the 5-, 10- and 15-year DSS rates were 62%, 53% and 27%, respectively. However, Mark Zupancic et al. [28] provided an opposite conclusion in a cohort study of ACC. Zupancic analyzed 142 ACC patients and found that PNI was not an independent influencing factor for disease-free survival (DFS) and overall survival (OS). And they discovered that there was no clear correlation between PNI and disease staging. In general, patients with PNI have a greater difficulty in achieving negative surgical margins and have a greater likelihood of LR and DM. The pathological findings of case 1 showed significant infiltration of tumor cells around the nerve and blood vessel walls, resulting to a relatively high probability of LR and DM.

According to the size of the lesion and the presence or absence of lymph node metastasis and DM, the clinical staging of case 1 is IVA stage. Brookstone MS [29] believed that PIACC should be divided into three stages based on the manifestation of bone destruction instead of tumor size. Stage I was defined as restricted intraosseous lesions with no changes in morphology and integrity of the bone cortex and the periosteum. Stage II was defined as expanded intraosseous lesions with an intact bone cortex. And Stage III was defined as expanded intraosseous lesions with damage of the bone cortex and the periosteum or lymph node metastasis. In case 1, the patient had a partial defect in the buccal bone cortex and perforation in the lingual bone cortex, with MRI examination confirming the presence of lymph node enlargement. According to the staging system of Brookstone MS, case 1 in our study was diagnosed as stage III. Tooth extraction in case 1 may accelerate the growth of the tumor and destruction of the bone cortex.

During the regular postoperative follow-up, seven patients experienced DM, three patients experienced LR and four patients passed away due to the tumor. Though the patient in case 1 has not shown LR or DM, a long-term follow-up is suggested.

## 4. Analysis of the Two Cases

Case 1 and case 2 showed generally similar characteristics when comparing the CT and MRI images of the two patients. Both cases presented osteolytic features centered on the mandible with ill-defined boundaries, while case 2 showed a mass with a center leaning more towards the lingual side, accompanied by invasion of surrounding soft tissues and the mandibular canal. In addition, damage of the bone cortex and periosteum can be noticed in both cases. But three-dimensional reconstruction images showed differences between the two patients. Case 1 demonstrated honeycomb-like bone resorption while case 2 demonstrated relatively regular and cystic bone resorption. The difference in three-dimensional reconstruction examinations may be a key point in distinguishing PIACC from RIACC, which needs more research to confirm. Both patients complained of paresthesia in the lower lip, with no obvious redness, swelling, hyperplasia or ulceration in the overlying mucus. And the pathological results showed a solid pattern in both cases. The two patients cannot be distinguished based on clinical and pathological characteristics. Medical history matters. When receiving patients in clinical practice, it is necessary to inquire about the medical history in detail.

Diagnostic criteria of PIACC were deemed as follows: (1) radiographic evidence of osteolysis, (2) intact cortical plates, (3) absence of any primary lesion within the salivary glands or other tissues that resemble the architecture of salivary lesions and (4) histologic confirmation of ACC [30]. Case 1 met these criteria excepting defection of bilateral cortical bone, indicating an aggressive growth pattern. On the contrary, case 2 did not meet the third criterion, which proved case 2 a recurrent lesion instead of a primary one.

ACC consists of ductal cells and myoepithelial cells. Myoepithelial cells have been shown to inhibit cell division and invasiveness, thereby playing a vital role in tumor growth inhibition [31]. Tumors comprising myoepithelial cells commonly have a low grade [32]. CD117 and CK7 are expressed in the lumen cells, while calponin, SMA, S-100 and p63 are myoepithelial markers. Immunohistochemical results like CD117 (+) and p63 (+) in case 1 show typical features of ACC. However, immunohistochemical results like calponin (−) and p63 (−) in case 2 indicate loss of myoepithelial differentiation, pointing out a high-grade transformation. As a proliferating cell nuclear protein, Ki-67 expression can accurately reflect the proliferative activity of tumor cells. Ki-67 (+, 30%) in case 2 refers to a high mitotic rate, also related to the high-grade transformation. It has been reported that a positive CD117 indicates low differentiation of myoepithelial cells, which results in a poor prognosis [33]. Similarly, the loss of S100 expression is correlated with a poor prognosis as well. Based on the study of Zheng Huang, the loss of S100 expression may be a prognostic factor of DM [34]. The younger age, late clinical stage and high pathological grade of case 2 imply a more aggressive ACC. But the late clinical stage and immunohistochemical results of case 1 imply a worse prognosis with a greater probability of DM. PIACC seems to be more malignant in our report. However, further research of immunohistochemical markers of IACC is needed.

For patients with LR, the most important consideration is the feasibility of local control. Ishida et al. [35] found that effective local control can improve the long-term survival rate of patients with LR. LR and lymph node metastasis occurred in the patient of case 2 14 years after initial surgery and PORT. Due to the unpredictability of DM, surgery remains the most effective means of achieving local control if the disease is amenable to local treatment measures. Surgery combined with PORT is still an ideal treatment option for patients with recurrence. Jeong et al. [36] found that patients with LR have a higher rate of DM. Because of the ill-defined boundary in case 2, simultaneous mandibular reconstruction was not recommended. However, in consideration of the patient’s insistence on restoring the shape and function of the mandible and the relatively young age of the patient, partial mandibulectomy accompanied by primary mandibular reconstruction with a fibular flap was finally adopted. Because the patient in case 2 already received radiotherapy totaling 70 Gy 14 years ago, it is not recommended to continue using PORT as an adjuvant treatment, due to the high possibility of complications after secondary radiotherapy. Moreover, if the disease inhibits local treatment measures, active systemic treatment is necessary, including particle radiotherapy, cytotoxic chemotherapy, targeted therapy, biological therapy and immunotherapy [37].

According to the study of Mark Zupancic [28], age, gender, smoking, PNI and radical surgery are not prognostic factors for disease-free survival and overall survival of ACC. Instead, patients of early clinical stages (stage I and II), patients of major salivary gland subsites and patients who have taken multimodal treatment (surgery and PORT) have the best prognosis. For the two patients of our report, late clinical stage (both stage IV) and high pathological grade predict poor prognosis, while PNI indicates high potential of DM. Other prognostic factors like immunohistochemical markers need to be further explored.

## 5. Conclusions

ACC mostly occurs in the three major glands and other minor glands in the oral cavity. PIACC is very rare with only 53 cases reported. IACC must be diagnosed based on clinical, radiological and histopathological manifestations. Detailed medical record collection plays an important role in distinguishing between primary and recurrent IACC. Whether it is PIACC or RIACC, treatment via surgical excision and PORT is the best way to achieve local control and to reduce the incidence of DM. Because of the high occurrence rate of DM, long-term follow-up is essential. Our report may provide a further understanding of the clinical, radiological and pathological features of IACC.

## Figures and Tables

**Table 1:** Data on 53 cases of primary intraosseous adenoid cystic carcinoma.

Number	Gender/Age (yr)	Site	Symptoms	Lymph Nodes Metastasis at First Visit	DM at First Visit	Surgery	Safety Margin	ND	Reconstruction	RT	CT	Histologic Type	Follow-Up
1	M/54	Body (R)	Pain, swelling	No	No	Partial mandibulectomy	/	No	No	Yes	No	Cribriform	Pulmonary metastasis
2	M/64	Angle (R)	Pain, swelling, trismus	No	No	Total mandibulectomy	Negative	No	Chrome–cobalt implant	No	No	/	/
3	F/40	Ramus	Swelling	No	Lung	No	/	No	No	No	No	/	/
4	M/35	Angle (R)	Swelling	No	No	Hemi-mandibulectomy	/	No	No	No	No	/	Lost to follow-up
5	F/50	Body (R)	Pain	No	No	Hemi-mandibulectomy	/	No	No	No	No	/	Pulmonary metastasis
6	F/47	Body (R)	Paresthesia, tooth mobility	No	The right Gasserian ganglion	No	/	No	No	Yes	No	Solid	/
7	M/47	Body (R)	Swelling, tooth mobility	No	No	Hemi-mandibulectomy	/	Yes	No	No	No	/	/
8	F/72	Body (L)	Pain	No	No	No/Palliative treatment	/	No	No	No	No	Cribriform	NED at 7 yr, lost to follow-up
9	F/68	Ramus (R)	Swelling	No	No	Partial mandibulectomy	Negative	No	No	Yes	No	Cribriform	Recurrence
10	M/82	Body, angle, ramus (L)	Pain, swelling	No	No	No	/	No	No	Yes	Yes	Cribriform	Died of pneumonia
11	F/33	Body, angle (R)	Pain, trismus	No	No	Hemi-mandibulectomy with preservation of the condyloid process	/	Yes	A free fibula flap and titanium plates	Yes	No	Cribriform	/
12	M/67	Body	/	No	No	Hemi-mandibulectomy	/	No	No	No	No	Cribriform, Solid	Lymph node metastases
13	F/46	Body (B)	Pain, swelling	No	No	Partial mandibulectomy	Negative	No	No	No	No	Cribriform	NED at 14 y
14	M/54	Body (R)	Swelling	Yes	No	Hemi-mandibulectomy	Negative	Yes	A free fibula flap and titanium plates	Yes	No	Cribriform	NED at 4.5 yr
15	M/51	Angle	/	/	/	Hemi-mandibulectomy	/	No	No	Yes	No	/	Died of the tumor at 4 yr
16	M/48	Body	/	/	/	Hemi-mandibulectomy	/	No	No	Yes	No	/	Died of the tumor at 3 yr
17	F/24	Angle	/	/	Lung	Hemi-mandibulectomy	/	No	No	Yes	No	/	AWD at 7 yr
18	F/35	Angle	/	/	/	Hemi-mandibulectomy	/	No	No	Yes	No	/	AWD at 5 yr
19	M/56	Body(R)	Pain	/	/	Apicoectomy Tooth extraction	/	No	No	No	No	Cribriform, tubular	lost to follow-up
20	F/36	Angle (R)	Pain, swelling, paresthesia	No	Lung	No	/	No	No	Yes	Yes	Cribriform	AWD at 3 yr
21	F/61	Angle (L)	Swelling	Yes	No	Partial mandibulectomy	Positive	Yes	Titanium plates	Yes	No	/	/
22	F/80	Body (L)	Pain	No	No	Partial mandibulectomy	/	No	No	No	No	Solid	NED at 3 yr
23	M/45	Body, angle (R)	Swelling	No	Lung	No	/	No	No	No	No	Solid	Died before treatment
24	F/57	Angle (L)	Paresthesia, tooth mobility	No	No	Partial mandibulectomy	Negative	No	No	Yes	No	Cribriform, tubular	NED at 3 yr
25	M/46	Body, angle, ramus (L)	Pain, paresthesia	No	Lung	Hemi-mandibulectomy	Negative	No	A free fibular flap and titanium plates	No	No	Cribriform, tubular	AWD at 2 yr
26	F/53	Body (B)	Pain, swelling, paresthesia	No	No	Partial mandibulectomy	/	No	Walter-Lorenz plate	No	No	Cribriform, tubular	NED at 3 yr
27	M/68	Body (B)	Swelling, paresthesia, tooth mobility	No	No	Partial mandibulectomy	Negative	Yes	A fibula free flap and titanium plates	Yes	Yes	Solid	Multiple bone metastases in the ribs, thoracic spine, pubis and pelvis 5 months after surgery. Died with lung metastases 13 months after first being seen
28	M/48	Body (L)	Pain	No	No	Hemi-mandibulectomy	/	No	No	Yes	No	Solid	/
29	F/45	Body (B)	Pain, paresthesia	No	No	Partial mandibulectomy	/	Yes	A fibula free flap and titanium plates	Yes	Yes	Cribriform	Pulmonary, breast, kidney and pancreatic metastases. Died from respiratory failure 7 years after curative surgery
30	M/64	Body (B)	Pain	No	No	Subtotal mandibulectomy (sparing the condylar stumps)	/	Yes	Titanium plates	No	No	Solid	NED at 15 mo
31	F/23	Body (R)	Pain, swelling	No	No	Hemi-mandibulectomy	Negative	No	A free fibular flap and titanium plates	Yes	No	Cribriform	NED at 10 mo
32	F/47	Body (B)	Swelling, paresthesia, tooth mobility	No	No	Partial mandibulectomy	/	No	A free iliac crest bone flap and titanium plates	No	No	Cribriform	NED at 6 mo
33	M/57	Body (L)	Pain, swelling, tooth mobility	No	No	Partial mandibulectomy	Negative	Yes	No	Yes	No	/	NED at 6 mo
34	F/42	Body	Pain, tooth mobility	No	No	Partial mandibulectomy	Negative	No	NO	Yes	No	Cribriform	NED at 6 mo
35	F/81	Body	Pain	Yes	No	Partial mandibulectomy	Negative	Yes	A pectoralis major musculocutaneous flap	No	No	Solid	Recurrence at 9 mo
36	F/46	Body	Swelling	No	No	Partial mandibulectomy	Negative	No	A free fibular flap and titanium plates	Yes	No	Cribriform	NED at 76 mo
37	F/74	Body	Pain	Yes	/	Partial mandibulectomy	Negative	Yes	A pectoralis major musculocutaneous flap	Yes	No	Solid	Died at 24 mo
38	M/66	Ramus	Swelling	No	No	Partial mandibulectomy	Negative	No	No	Yes	No	Tubular	NED at 21 mo
39	F/63	Body	Swelling	No	No	Partial mandibulectomy	Negative	No	No	Yes	No	Cribriform	Recurrence at 144 mo
40	F/28	Body	Paresthesia	Yes	No	Partial mandibulectomy	Negative	Yes	A radial forearm free flap	Yes	No	Tubular	NED at 42 mo
41	M/65	Body	Swelling	No	No	Partial mandibulectomy	Negative	No	No	Yes	No	Cribriform	NED at 74 mo
42	F/57	Body, angle (R)	Swelling, paresthesia	No	No	Partial mandibulectomy	/	No	No	Yes	Yes	Cribriform	Pulmonary metastasis 3 months after surgery. AWD at 3 yr
43	M/41	Body, angle, ramus (R)	Toothache, swelling, paresthesia, trismus	No	No	No	/	No	No	No	No	Solid	bone and lymph node metastases 1 year after biopsy. AWD at 1 yr
44	M/58	Body (L)	Pain, paresthesia	No	No	Partial mandibulectomy	/	No	No	Yes	No	Solid	NED at 5 mo
45	M/54	Body (L)	Swelling	No	No	Partial mandibulectomy	/	No	No	Yes	No	Cribriform	NED at 3 mo
46	F/38	Body (L)	Pain, swelling	Yes	No	Hemi-mandibulectomy	Negative	Yes	A free rib skin flap and titanium plates	Yes	No	Cribriform	NED at 2 yr
47	M/52	TMJ area (L)	Pain, trismus	Yes	Lung	No	/	No	No	No	Yes	Cribriform	AWD at 2 yr
48	F/71	Body (B)	Pain, swelling	No	No	Partial mandibulectomy	/	No	No	Yes	No	Cribriform	NED at 15 mo
49	M/55	Body (L)	Swelling, pain	No	No	Hemi-mandibulectomy	Negative	No	A free fibular flap and titanium plates	No	No	Solid	Died of the disease within 3 years
50	F/54	Angle, ramus (R)	swelling, pain, paresthesia	Yes	Bone	No/Palliative treatment	/	No	No	No	No	Solid	/
51	M/49	Body, angle (R)	Swelling	/	/	No	/	No	No	No	No	Solid, cribriform	lost to follow-up
52	F/70	Body, angle, ramus (R)	paresthesia	Yes	Bone	No	/	No	No	Yes	Yes	Solid	AWD at 3 yr
53	F/65	Body, angle, ramus (R)	Pain, swelling, paresthesia	Yes	No	Hemi-mandibulectomy(R) Partial mandibulectomy(L)	Negative	Yes	An anterolateral thigh perforator flap	Yes	No	Solid	NED at 15 mo

Abbreviations: F, female; M, male; R, right; L, left; B, bilateral; ND, neck dissection; RT, radiotherapy; CT, chemotherapy; AWD, alive with disease; NED, no evidence of disease; TMJ, temporomandibular joint.

**Table 2:** Literature review on initial diagnosis of 31 cases.

Initial Diagnosis	Total, *n* (%)
Ameloblastoma	7 (22.6%)
Odontogenic infection or cyst	11 (35.5%)
Periapical cemental dysplasia	1 (3.2%)
Malignant tumor	12 (38.7%)

## Data Availability

Data are contained within the article.
